# Selective Involvement of the Amygdala in Systemic Lupus Erythematosus

**DOI:** 10.1371/journal.pmed.0030499

**Published:** 2006-12-19

**Authors:** Bart J Emmer, Jeroen van der Grond, Gerda M Steup-Beekman, Tom W. J Huizinga, Mark A van Buchem

**Affiliations:** 1 Department of Radiology, Leiden University Medical Center, Leiden, The Netherlands; 2 Department of Rheumatology, Leiden University Medical Center, Leiden, The Netherlands; Columbia University, United States of America

## Abstract

**Background:**

Antibodies specifically affect the amygdala in a mouse model of systemic lupus erythematosus (SLE). The aim of our study was to investigate whether there is also specific involvement of the amygdala in human SLE.

**Methods and Findings:**

We analyzed a group of 37 patients with neuropsychiatric SLE (NP-SLE), 21 patients with SLE, and a group of 12 healthy control participants with diffusion weighted imaging (DWI). In addition, in a subset of eight patients, plasma was available to determine their anti-NMDAR antibody status. From the structural magnetic resonance imaging data, the amygdala and the hippocampus were segmented, as well as the white and gray matter, and the apparent diffusion coefficient (ADC) was retrieved. ADC values between controls, patients with SLE, and patients with NP-SLE were tested using analysis of variance with post-hoc Bonferroni correction. No differences were found in the gray or white matter segments. The average ADC in the amygdala of patients with NP-SLE and SLE (940 × 10^−6^ mm^2^/s; *p* = 0.006 and 949 × 10^−6^ mm^2^/s; *p* = 0.019, respectively) was lower than in healthy control participants (1152 × 10^−6^ mm^2^/s). Mann-Whitney analysis revealed that the average ADC in the amygdala of patients with anti-NMDAR antibodies (*n* = 4; 802 × 10^−6^ mm^2^/s) was lower (*p* = 0.029) than the average ADC of patients without anti-NMDAR antibodies (*n* = 4; 979 × 10^−6^ mm^2^/s) and also lower (*p* = 0.001) than in healthy control participants.

**Conclusions:**

This is the first study to our knowledge to observe damage in the amygdala in patients with SLE. Patients with SLE with anti-NMDAR antibodies had more severe damage in the amygdala compared to SLE patients without anti-NMDAR antibodies.

## Abstract 2

Patients with SLE who also had antibodies against the NMDA receptor had more severe damage in the amygdala as compared with patients with SLE without these antibodies.

## Editors' Summary

**Background.:**

The human body is continually attacked by viruses, bacteria, fungi, and parasites, but the immune system usually prevents these pathogens from causing disease. To be effective, the immune system has to respond rapidly to foreign antigens (bits of proteins that are unique to the pathogen) but ignore self-antigens. In autoimmune diseases, this ability to discriminate between self and nonself fails for unknown reasons, and the immune system begins to destroy human tissues. In the chronic autoimmune disease systemic lupus erythematosus (SLE or lupus), the immune system attacks the skin, joints, nervous system, and many other organs. Patients with SLE make numerous “autoantibodies” (antibodies are molecules made by the immune system that recognize and attack antigens; autoantibodies attack self-antigens). These autoantibodies start the attack on the body; then other parts of the immune system join in, causing inflammation and forming deposits of immune cells, both of which damage tissues. Common symptoms of SLE include skin rashes and arthritis, but some patients develop NP-SLE, a form of SLE that includes neuropsychiatric symptoms such as amnesia, dementia, mood disorders, strokes, and seizures. There is no cure for SLE, but mild cases are controlled with ibuprofen and other non-steroidal anti-inflammatory drugs; severe cases are kept in check with corticosteroids and other powerful immunosuppressants.

**Why Was This Study Done?:**

In most of the tissues affected by SLE, the damage done by autoantibodies and immune cells can be seen when the tissues are examined with a microscope. But there is little microscopic damage visible in the brains of patients with NP-SLE. More generally, it is unclear how or even whether the immune system affects mental functions and emotion. In this study, researchers used magnetic resonance imaging (MRI) to investigate whether there are any structural changes in the brains of patients with NP-SLE that could explain their neuropsychiatric symptoms. They have also examined whether any changes in the brain can be linked to the presence of autoantibodies that recognize a protein called the NMDA receptor (anti-NMDAR antibodies) that is present on brain cells.

**What Did the Researchers Do and Find?:**

The researchers used an MRI technique called diffusion weighted imaging to examine the brains of several patients with NP-SLE or SLE and the brains of several healthy individuals. Using this technique, it is possible to quantify the amount of structural damage in different regions of the brain. The researchers found no differences in most areas of the brain between the two groups of patients and the healthy controls. However, there were clear signs of damage in the amygdala (the part of the brain that regulates emotions and triggers responses to danger) in the patients with SLE or NP-SLE when compared to the control individuals. The researchers also found that the damage was more severe in the patients who had anti-NMDAR autoantibodies than in those that did not have these autoantibodies.

**What Do These Findings Mean?:**

These findings suggest that autoantibodies produced by patients with SLE specifically damage the amygdala, a discovery that helps to explain some of the neuropsychiatric symptoms of this condition. Previous work has shown that the treatment of mice with anti-NMDAR antibodies and epinephrine, a stress hormone that causes leaks in the blood-brain barrier (antibodies can't usually get into the brain because of this barrier), results in damage to the amygdala and a deficient response to dangerous stimuli. The researchers suggest that a similar series of events might happen in SLE—patients often mention that a period of major stress precedes the development of symptoms. To provide stronger evidence for such a scenario, a detailed study of how stress relates to neuropsychiatric symptoms is needed. The damage to the amygdala (and the lack of damage elsewhere in the brain) and the possible association between brain damage and anti-NMDAR antibodies seen in this small study also need to be confirmed in more patients. Nevertheless, these findings provide an intriguing glimpse into the interplay between the immune system and the brain and into how stress might lead to physical damage in the brain.

**Additional Information.:**

Please access these Web sites via the online version of this summary at http://dx.doi.org/10.1371/journal.pmed.0030499.

- MedlinePlus encyclopedia pages on autoimmunity and on systemic lupus erythematosus
- US National Institute of Arthritis and Musculoskeletal and Skin Diseases booklet for patients with SLE
- American College of Rheumatology information for patients on SLE
- NHS Direct Online Health Encyclopedia pages on SLE
- The Lupus Foundation of America information and support for patients with SLE

## Introduction

The influence of the immune system on cognition and emotion is unclear. Recently, it was shown that antibodies could alter emotional behavior in a rodent model of human autoimmune disease, systemic lupus erythematosus (SLE) [1]. SLE is characterized by the production of various types of autoantibodies; it is the autoimmune disease with the largest number of detectable autoantibodies [2,3]. The most specific autoantibody present in the serum of patients with SLE is directed against DNA. Neuropsychiatric (NP) symptoms can occur in SLE patients, and these patients are classified as having NP-SLE. These NP symptoms can be divided into primary, caused by SLE, and secondary to comorbidity in SLE. The rheumatology department of our institution, which serves an area of roughly 2 million inhabitants, reported primary NP-SLE in 30 (15.7%) of 191 patients with SLE, using data accumulated over a 10-y period [4].

The origin of primary NP symptoms in patients with SLE has long been a mystery, because the scarce histological material obtained from such patients failed to provide clues for interactions between autoantibodies and the brain. Moreover, it has become clear that different pathogenic pathways can lead to neurological symptoms in patients with SLE [5]. Patients with SLE may have autoantibodies, which interfere with blood clotting, leading to brain infarctions. SLE patients may also suffer from neurological manifestations that are presumably caused by antibodies binding to neural cells [6–8].

Previously, it has been demonstrated that a subset of the antibodies to double stranded DNA (dsDNA) in patients with SLE cross-reacts with subunits of the NMDA receptor (anti-NMDAR antibodies) on neuronal cells and can cause neuronal death by excito-toxicity and apoptosis [7,9]. Under normal circumstances, the blood-brain barrier (BBB) prevents these antibodies from causing neuronal damage. By using bacterial lipopolysaccharide to breach the BBB, brain damage was induced in mice with anti-NMDAR antibodies. In that model, the hippocampus was preferentially affected [10]. The same mouse model was used to assess whether rises in epinephrine, a stress hormone which is known to cause leaks in the BBB, could also induce brain damage in the presence of anti-NMDAR antibodies. These animals developed a behavioral disorder characterized by a deficient response to fear-conditioning paradigms. Symptoms could be explained by the observed selective neuronal loss in the amygdala, a structure that is part of the limbic system and is involved in regulating emotions such as stress, fear, and depression [1].

The aim of our study was to assess whether the hippocampus and the amygdala are selectively affected in patients with NP-SLE and SLE, and whether anti-NMDAR antibodies are involved in creating changes in these brain structures.

## Methods

### Patients

We obtained informed consent from all patients and controls, and the hospital's commission on scientific research on human subjects approved the study protocol.

All patients with SLE fulfilled the 1982 American College of Rheumatology (ACR) revised criteria for SLE [11]. The patients with SLE had an average SLE disease duration of 4.2 y (SD 4.9). None of the patients with SLE had a history of or active neurological disease at the time of the scan.

Healthy controls were recruited through advertisement in a local newspaper. Twelve healthy controls (1 male; 11 female; mean age 43.8 y; SD 9.5) were included in the study. Healthy controls were age and sex matched to the general characteristics of the patient population. Predefined exclusion criteria for control participants were a history of neurological disease or pathology on T1- or T2-weighted magnetic resonance imaging (MRI) scans.

Thirty-seven patients (1 male; 36 female; mean age 36.4 y; SD 13.0) were diagnosed as having NP-SLE according to the 1999 American College of Rheumatology revised criteria [12]. NP-SLE was diagnosed based on clinical symptoms. The following neuropsychiatric syndromes were present in our NP-SLE patient group: Guilian-Barre (*n* = 1), cerebrovascular disease (*n* = 11), headache (*n* = 12), mononeuropathy (*n* = 2), movement disorder (*n* = 3), myelopathy (*n* = 3), cranial neuropathy (*n* = 1), plexopathy (*n* = 2), seizures (*n* = 10), acute confusional state (*n* = 2), anxiety disorder (*n* = 1), cognitive dysfunction (*n* = 9), mood disorder (*n* = 5), and psychosis (*n* = 1). There were 20 patients with one syndrome, ten patients with two syndromes, five patients with three syndromes, and two patients with four syndromes. Special care was taken to exclude any other possible causes of NP symptoms, so that only patients with primary NP-SLE were included in the group [12]. There was no indication of other previous neurological or psychiatric disease in any of the participants. The patients with NP-SLE had an average SLE disease duration of 9.4 y (SD 8.8) and a history of neuropsychiatric involvement for an average of 4.5 y (SD 5.3). At the time of the scan, 11 patients had active disease defined as having had symptoms up to 6 mo before the scan. The remaining 26 patients had inactive disease, defined as having had no symptoms for at least 6 mo.

In addition, plasma was available to determine the anti-NMDAR antibody status in a subset of eight patients [7] (courtesy of Betty Diamond, Department of Medicine, Columbia University Medical Center, New York, United States). Autoantibodies to a linear epitope of the NR2 subunit of the NMDA receptor were assessed in eight patients by enzyme-linked immunosorbent assay (ELISA) using 96-well microtiter plates. In each assay, five negative control sera were included. The plates were read after 90 min and optical density (OD) was monitored at 405 nm. The anti-peptide antibody ELISA was performed as described previously by Putterman and Diamond [13]. Patients were considered to be anti-NMDAR antibody–positive based on the cut-off value of 2 standard deviations above the mean OD of the control sera.

Mean OD values (±SD) in NMDA-positive patients were 0.469 (±0.103, range 0.382–0.609) and 0.329 (±0.108, range 0.173–0.405).

In the group of anti-NMDAR antibody–positive patients, only one patient was anti-dsDNA antibody–positive (anti-dsDNA titers were measured at the time of the diffusion weighted imaging [DWI] scan). All anti-NMDAR antibody–negative patients were anti-dsDNA antibody–negative. This finding is in line with the previous study of Husebye showing no association between the anti-dsDNA antibodies and anti-NMDAR antibodies [14].

The patients with NP-SLE received one or more of the following medications for their NP symptoms at the time of scan: methylprednisolone (*n* = 11), cyclophosphamide (*n* = 6), azothioprane (Imuran) (*n* = 10), prednisone (*n* = 12), carbasalate calcium (Ascal) (*n* = 8), and fenprocoumon (Marcoumar) (*n* = 7). One patient underwent plasmapheresis and received intravenous immunoglobulin therapy. Eight patients were without any medication for their NP symptoms at the time of the scan. The majority of the patients with SLE had been treated with corticosteroids prior to the MRI scan. However, as recently demonstrated, DWI parameters are not influenced by oral corticosteroids [15].

### Imaging

All patients underwent DWI, an MRI technique that is particularly sensitive to structural brain damage, in which the apparent diffusion coefficient (ADC) is a measure reflecting tissue integrity in a quantitative way. Scan-rescan reproducibility of the mean ADC values has previously been shown to be robust [16]. The DWI consisted of a multishot spin-echo echo planar imaging (EPI) sequence, with an EPI factor defined as the number of rows in K-space collected per excitation, of 15. The total echo time was 114 ms. Other parameters were as follows: 256 × 128 matrix, 20 axial sections of 6 mm with an intersection gap of 1 mm, and a field of view of 230 mm covering the whole brain. The *b* factor was 800 s/mm^2^ applied to measure diffusion in three orthogonal directions. The maximum gradient strength of the machine was 23 mT/m. The slew rate of the system was 105 T/m/sec with a rise time of 0.22 s. From the DWI images in each of the three orthogonal directions, an average DWI was calculated. The ADC maps of the whole brain were calculated from the average DWI and *b*_0_ images on a voxel-by-voxel basis.

### Post-processing

**Figure 1:**
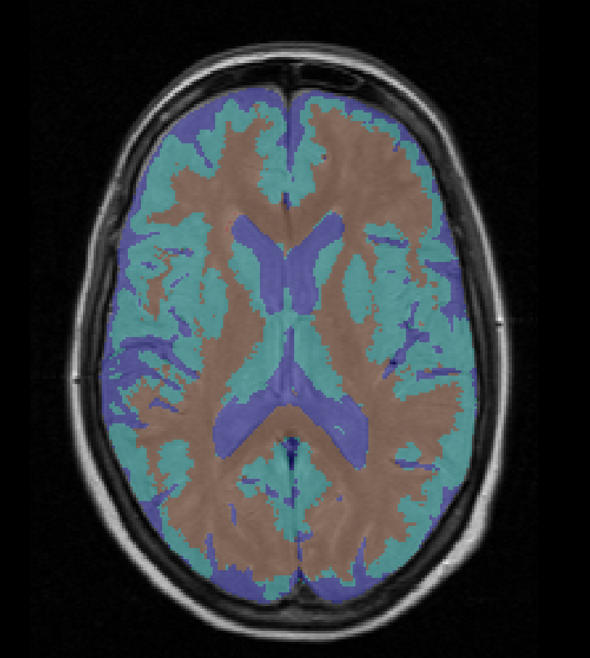
Axial Calculated Magentization Transfer Ratio Image Showing Segmentation of Cebrebrospinal Fluid (Dark Blue), the Gray Matter (Turquoise), and the White Matter (Brown)

**Figure 2:**
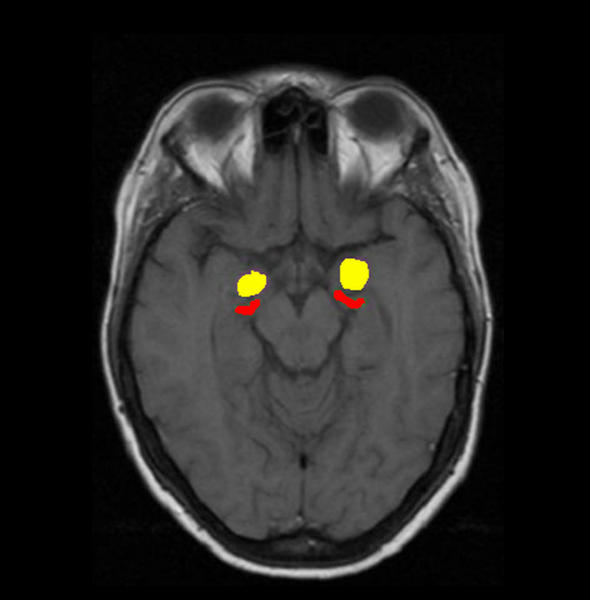
Axial T1 Weighted Anatomical MRI Scan Showing Segmentation of the Amygdala (Yellow) and the Hippocampus (Red)

We automatically segmented the cortical gray matter and white matter using Software for Neuro-Image Processing in Experimental Research (SNIPER), an in-house–developed program for image processing (Figure 1) [17]. In addition, we manually segmented regions of interest (ROIs) on coregistered T1 weighted images in the amygdala and in the hippocampus (Figure 2), on which the clinical status of the patient had been hidden. These ROIs subsequently mapped on the ADC maps. The average ADC was calculated for the ROIs, the white matter, and the gray matter. Macroscopic lesions were not included in the ROI.

### Statistical Analysis

Average ADC values from white and gray matter as well as from the ROIs were compared between controls and patients with NP-SLE using ANOVA analysis with post-hoc Bonferroni correction. An exact *p* value lower than 0.05 was considered significant. To test for differences between the controls and the anti-NMDAR– positive and –negative patients, nonparametric Mann-Whitney tests were used to account for differences in group size as well as small sample size.

## Results

The ADC values of gray matter, white matter, hippocampus, and amygdala in controls, patients with NP-SLE, and patients with SLE are shown in Table 1. No difference in the gray matter, white matter, or the hippocampus was found between groups. In patients with SLE (*p* = 0.019) as well as in patients with NP-SLE (*p* = 0.006), the ADC was decreased in the amygdala compared to controls. There was no difference in ADC values of the amygdala between patients with SLE and those with NP-SLE.

**Table 1:**
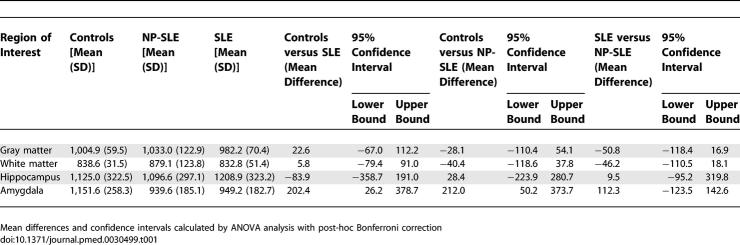
Mean ADC Values (×10^−6^ mm^2^/s) and Standard Deviations for all Participants

Table 2 shows ADC values of the hippocampus and the amygdala in control participants, anti-NMDAR–negative NP-SLE patients, and anti-NMDAR–positive NP-SLE patients. In patients with anti-NMDAR antibodies, the ADC was decreased (*p* = 0.001) compared to the healthy controls, whereas this was not the case (*p* = 0.262) for the patients without the anti-NMDAR antibodies. In addition, the ADC in anti-NMDAR–positive patients is decreased (*p* = 0.029) compared to patients without these antibodies.

**Table 2:**
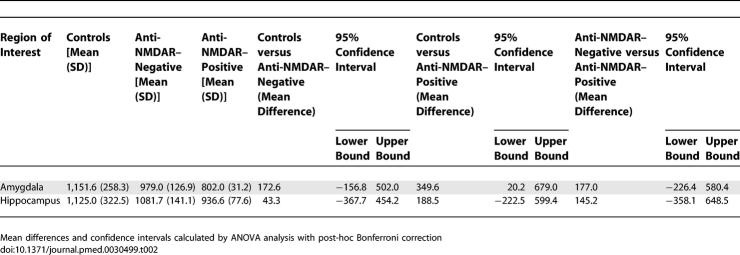
Mean ADC Values (×10^−6^ mm^2^/s) and Standard Deviations for Controls, Anti-NMDAR–Positive and –Negative Participants

## Discussion

This is the first study to our knowledge to observe selective damage in the amygdala in patients with SLE. In contrast, we did not find significant changes in ADC of the white matter, gray matter, or the hippocampus. These findings indicate that the amygdala is specifically affected by the autoantibodies and also suggests that the animal model in which the BBB is opened by increased cerebral blood flow induced by a stress hormone could be an appropriate reflection of human disease. Although the sample size is small, we observed more severe changes in patients with SLE with anti-NMDAR antibodies as compared to patients with SLE without anti-NMDAR antibodies, suggesting that these antibodies induce brain damage. The low ADC in the amygdala is compatible with the presence of cytotoxic edema [18].

The finding that the amygdala in patients with SLE is significantly different from that in healthy controls is in line with the report in the mouse model of SLE, showing that antibodies can affect the limbic system, which can result in altered emotions [1]. Usually, animal models of human diseases are only an approximation of actual disease in humans. However, the finding that the amygdala is selectively involved and that this involvement was more pronounced in patients with anti-NMDAR antibodies than in patients without these antibodies supports the validity of this mouse model.

Epinephrine is released under circumstances of stress, and patients with SLE often relate the occurrence of major stress to the induction of organ involvement. Although epidemiological data are currently lacking for a correlation between episodes of stress and the development of NP symptoms in SLE patients, such a relation could explain our data. Furthermore, in the mouse model, the stress hormone epinephrine opened the BBB at the site of the amygdala. This observation would also explain the selective involvement of the amygdala compared to the residual brain tissue in our patients.

A limitation of our study is the small number of participants. Given the different pathogenetic pathways leading to NP symptoms in patients with SLE, such as those secondary to lupus nephritis or mediated by anti-phospholipid antibodies, we took great care in patient selection that only patients in whom extensive work-up revealed that the symptoms were most likely to be caused by primary SLE or NP-SLE were included. As mentioned earlier, the number of new patients with primary SLE or NP-SLE referred to a tertiary referral center such as ours over a long period of time is not substantial [4]. Hence, there are not a large number patients available to study. Further, the number of patients eligible for anti-NMDAR determination is also limited. However, the effects measured in the amygdala are consistent and in contrast with the trends for increased ADC values found in the remaining brain tissue. Still, we recognize that for the clinical validation of our findings, a much larger sample will be required. Another limitation of our study could be the relatively small ROI drawn in the hippocampus; this limitation occurs because of the axial orientation of the scan slices, which is not the ideal orientation for hippocampal segmentation. Nonetheless, this limitation has no influence on the findings in the amygdala, although further studies using coronal slices for more extensive hippocampal segmentation could possibly reveal effects in the hippocampus as well.

Our observations provide an insight into the interplay of the immune system on the one hand and cognition and emotion on the other. The immune system, through the generation of autoantibodies that cross-react with neuronal receptors, can cause damage of specific brain structures resulting in specific types of cognitive and/or emotional changes. Alternatively, emotions may render specific brain structures more vulnerable through increased secretion of stress hormones that breach the BBB in specific brain areas. This is, to our knowledge, the first example of the elucidation of a pathogenetic mechanism by which major stress could lead to an organic brain syndrome.

## Abbreviations

ADC: apparent diffusion coefficient
BBB: blood-brain barrier
DWI: diffusion weighted imaging
NP: neuropsychiatric
OD: optical density
ROI: region of interest
SLE: systemic lupus erythematosus
